# Temporal trends and factors associated with increased mortality among atrial fibrillation weekend hospitalizations: an insight from National Inpatient Sample 2005–2014

**DOI:** 10.1186/s13104-019-4440-8

**Published:** 2019-07-12

**Authors:** Dinesh C. Voruganti, Ghanshyam Shantha, Sushma Dugyala, Naga Venkata K. Pothineni, Deobrat Chandra Mallick, Abhishek Deshmukh, Ala Mohsen, Stephanie S. Colello, Mohammed Saeed, Rakesh Latchamsetty, Krit Jongnarangsin, Frank Pelosi, Ryan M. Carnahan, Michael Giudici

**Affiliations:** 1 Division of Internal Medicine, Roy and Lucille J. Carver College of Medicine, University of Iowa Hospitals and Clinics, 200 Hawkins Dr., Internal Medicine, Iowa City, IA 52242 USA; 2 Division of Electrophysiology, University of Michigan, Ann Arbor, MI USA; 3 Division of Internal Medicine, University of Alabama, Tuscaloosa, USA; 4 Division of Cardiovascular Medicine, University of Arkansas, Little Rock, AR USA; 5 Department of Hospital Medicine, Spohn Shoreline Hospital, Corpus Christi, TX USA; 6 Division of Cardiovascular Medicine, Mayo Clinic, Rochester, MN USA; 7 Division of Cardiovascular Medicine, Louisiana State University, New Orleans, LA USA; 8 Division of Internal Medicine, Hospital of the University of Pennsylvania, Philadelphia, PA USA; 9 College of Public Health, University of Iowa, Iowa City, IA USA; 10 Division of Cardiovascular Medicine, Electrophysiology, Roy and Lucille J. Carver College of Medicine, University of Iowa Hospitals and Clinics, Iowa City, IA USA

**Keywords:** Atrial fibrillation, Weekend hospitalization, In-hospital mortality

## Abstract

**Objective:**

Atrial fibrillation (AF) weekend hospitalizations were reported to have poor outcomes compared to weekday hospitalizations. The relatively poor outcomes on the weekends are usually referred to as ‘weekend effect’. We aim to understand trends and outcomes among weekend AF hospitalizations. The primary purpose of this study is to evaluate the trends for weekend AF hospitalizations using Nationwide Inpatient Sample 2005–2014. Hospitalizations with AF as the primary diagnosis, in-hospital mortality, length of stay, co-morbidities and cardioversion procedures have been identified using the international classification of diseases 9 codes.

**Results:**

Since 2005, the weekend AF hospitalizations increased by 27% (72,216 in 2005 to 92,220 in 2014), mortality decreased by 29% (1.32% in 2005 to 0.94% in 2014), increase in urban teaching hospitalizations by 72% (33.32% in 2005 to 57.64% in 2014), twofold increase in depression and a threefold increase in the prevalence of renal failure were noted over the period of 10 years. After adjusting for significant covariates, weekend hospitalizations were observed to have higher odds of in-hospital mortality OR 1.17 (95% CI 1.108–1.235, P < 0.0001). Weekend AF hospitalizations appear to be associated with higher in-hospital mortality. Opportunities to improve care in weekend AF hospitalizations need to be explored.

## Introduction

Atrial fibrillation (AF), the most common sustained arrhythmia in clinical practice had an estimated worldwide prevalence of 33.5 million in 2010 [1]. AF weekend hospitalizations were previously reported to have higher mortality and lower rates of cardioversion [2]. Subsequent studies in this population have demonstrated improved mortality and rates of cardioversion [3, 4]. To date, there has been no temporal trend analysis showing this effect. We sought to investigate the outcomes in the years 2005–2014 through a publicly available national inpatient sample database (NIS).

## Main text

### Methods

A description of NIS database has been elaborated in prior studies [5–7]. The NIS is one of the largest, all-payer database for the United States in-patient hospitalizations, and it is maintained by the Agency for Health Care Quality and Research (AHRQ). The NIS includes a 20% random sample of all inpatient hospitalizations from 46 states in the United States. Each observation represents a hospitalization with one primary diagnosis, up to 29 secondary diagnoses and 15 procedure diagnosis with International Classification of Disease, 9th revision, clinical modification (ICD-9-CM) codes.

NIS hospitalizations have 2 sampling strategies. Before 2012, all hospitalizations were from a random sample of 20% of acute care hospitals in the United States, stratified by bed size, region, and location. Starting in 2012, the NIS included a random sample of 20% of discharges from all acute care hospitals in the United States; this effort reduced the margin of error by 50%, and national estimates decreased by 4.3%. From 1998 to 2011, discharge weights are provided by the AHRQ after a validation process, and they are used to calculate national estimates. To account for changes in the sampling strategies, the variable “trend weights” have been used for 2011 and all preceding years to facilitate trend analysis from 1998 to 2014 as recommended by AHRQ [8].

The study was exempted by the University of Iowa, Iowa City, institutional review board as it includes only de-identified, publicly available data. For our analysis, we only used NIS data from 2005 to 2014. Similar to previous studies, we used the ICD-9-CM code 427.31 to identify hospitalizations involving hospitalizations with principal diagnosis (dx1) of AF [9]. The variables for hospitalization demographics were provided in the dataset (example: age, gender, length of stay). The weekend hospitalizations (Saturday–Sunday) were identified using ‘AWEEKEND’ variable. Hospitalizations with anticoagulation were identified using the ICD-9-CM code ‘V58.61’. ICD-9-procedure codes 9961, 9962, 9969 and 3734 were used to identify hospitalizations with cardioversion/ablation.

We used survey analysis methods to account for the clustering and stratification of encounters for all continuous and categorical variables. SAS 9.4 (SAS Institute Inc., Cary, North Carolina) software were used to perform statistical analysis. We used sampling weights to estimate trends and national estimates to account for the change in sampling design as recommended by the AHRQ. For the demographics, co-morbid diseases, and weekend hospitalizations within each year were compared using Student’s *t* test for continuous variables and the Chi square test for categorical variables. Multivariate logistic regression method was used in SAS (*proc surveylogistic*) to evaluate the association between weekend hospitalizations and in-hospital mortality after including the other variables for potential confounders. C-statistic was used for goodness of the model fit for a binary outcome. Like previous studies, trends in demographics, co-morbid diseases, weekend hospitalizations involving AF hospitalizations, length of hospitalization, in-hospital mortality were evaluated using the *survey logistic* models after creating dummy variables for each outcome of interest. A P-value < 0.05 was considered statistically significant. The checklist provided by NIS was used to ensure the appropriateness of data analysis as recommended by AHRQ [10].

### Results

From January 2005–December 2014, we identified 4,520,409 weighted national estimated AF hospitalizations from NIS 2005–2014 database. Of these, there were 874,944 weekend hospitalizations. The mean age (years) ± standard deviation was slightly lower in the weekday group vs. the weekend group (69.85 ± 13.90 vs. 70.02 ± 14.74, P < 0.0001). The AF weekend group had lower elective admissions, relatively higher female hospitalizations, significantly lower utilization rates of cardioversion (14.17% vs. 23.62%, P < 0.0001), and lower cost of hospitalization (mean USD) 7479 vs. 8414 (p < 0.0001) (Table 1).

**Table 1:** Demographic characteristics of hospitalizations with atrial fibrillation on weekend and weekday

	AF on weekday	AF on weekend	P value
Unweighted index admissions	752,845	180,573	
Weighted index admissions	3,645,465	874,944	
Age in years at admission			
Mean age (in years), standard deviation	69.85, 13.90	70.02, 14.74	< 0.0001
18 to 34	1.48%	2.08%	< 0.0001
35 to 49	6.54%	7.37%	
50 to 64	24.39%	22.73%	
65 to 79	39.63%	37.23%	
Greater than 80	27.85%	30.49%	
Died during hospitalization			< 0.0001
Did not die	99.11%	98.92%	
Died	0.89%	1.08%	
Disposition			< 0.0001
Routine	76.44%	72.63%	
Transfer to short-term hospital	2.37%	2.73%	
Transfer other: includes Skilled Nursing Facility (SNF), Intermediate Care Facility (ICF), and another type of facility	9.51%	11.94%	
Home Health Care (HHC)	10.01%	10.55%	
Against medical advice (AMA)	0.76%	1.05%	
Died in hospital	0.89%	1.08%	
Discharged alive, destination unknown	0.02%	0.02%	
Elective vs. non-elective admission			< 0.0001
Non-elective	82.70%	95.35%	
Elective	17.30%	4.65%	
Indicator of sex			< 0.0001
Male	50.26%	47.74%	
Female	49.74%	52.26%	
Length of hospital stay			
Mean length of stay (days) ± standard deviation	3.48 ± 3.72	3.52 ± 3.79	< 0.0001
0–3 days	65.66%	64.67%	< 0.0001
4 to 6 days	22.56%	25.13%	
7 to 9 days	7.45%	5.38%	
10 to 12 days	2.15%	2.84%	
> 12 days	2.15%	1.98%	
Primary expected payer			< 0.0001
Medicare	65.49%	81.93%	
Medicaid	4.08%	8.25%	
Private insurance	25.29%	5.76%	
Self-pay	2.83%	1.47%	
No charge	0.32%	0.46%	
Other	1.99%	2.13%	
Race			< 0.0001
White	83.39%	81.93%	
Black	7.49%	8.25%	
Hispanic	5.13%	5.76%	
Asian	1.35%	1.47%	
Pacific Islander	0.51%	0.46%	
Other	2.13%	2.13%	
Cost of hospitalization in USD-(mean)	8414.7 ± 10,343	7479 ± 8785.9	< 0.0001
Bed size of the hospital			< 0.0001
Small	13.52%	14.27%	
Medium	24.91%	26.12%	
Large	61.57%	59.61%	
Location/teaching status of the hospital			< 0.0001
Rural	13.62%	14.80%	
Urban-non-teaching	41.30%	43.86%	
Urban-teaching	45.08%	41.34%	
Cardioversion	23.62%	14.17%	< 0.0001

Univariate and multivariate logistic regression analyses were performed. In multivariate analysis, the weekend hospitalizations were associated with higher odds of in-hospital mortality (OR 1.170, 95% CI 1.108–1.125, P < 0.0001) (Table 2). Apart from the weekend admission status, acute respiratory failure, congestive heart failure, renal failure and urban hospital admission (teaching and non-teaching) were found to be strong predictors of in-hospital mortality.

**Table 2:** Multivariate logistic regression analysis showing the adjusted odds ratio’s predicting the in-hospital mortality for atrial fibrillation (AF) hospitalizations

Effect	Odds ratio	95% confidence limits		P value
Weekend hospitalization	1.170	1.108	1.235	< 0.0001
Length of stay	1.026	1.02	1.031	< 0.0001
AGE	1.054	1.052	1.057	< 0.0001
Hospital region-north east	Reference group			
Hospital region-midwest	0.729	0.673	0.791	< 0.0001
Hospital region-south	0.885	0.825	0.95	7E−04
Hospital region-west	0.791	0.723	0.865	< 0.0001
Hypertension	0.569	0.542	0.596	< 0.0001
Uncomplicated diabetes	0.975	0.92	1.034	0.401
Complicated diabetes	1.194	1.067	1.338	0.002
Congestive heart failure	1.668	1.354	2.054	< 0.0001
Valvular heart disease	1.305	0.97	1.755	0.079
Renal failure	1.957	1.847	2.075	< 0.0001
Obesity	0.641	0.585	0.703	< 0.0001
Female sex	0.937	0.894	0.982	0.007
Small sized hospital	Reference group			
Medium sized hospital	1.11	1.023	1.204	0.012
Large sized hospital	1.092	1.015	1.176	0.018
Rural hospital	Reference group			
Urban non-teaching hospital	0.861	0.799	0.927	< 0.0001
Urban teaching hospital	0.887	0.823	0.955	0.002
Acute respiratory failure	21.2	19.997	22.476	< 0.0001

The adjusted odds ratio’s, 95% confidence intervals and their P-values represent the odds of in-hospital mortality after adjusting for the covariates listed in the table

Over the period of 10 years, we noticed increasing number of weekend hospitalizations with AF (72,216 in 2005 to 92,220 in 2014) (Table 3). In-hospital mortality has gradually decreased (1.32% in 2005 vs 0.94% in 2014, P trend < 0.0001), decreasing mean LOS (3.66 days in 2005 to 3.49 days in 2014, P trend < 0.0001), higher prevalence of depression (5.47% in 2005 vs 9.72% in 2014, P trend < 0.0001), increased rates of cardioversion (11.49% in 2005 vs 17.34% in 2014, P trend < 0.0001), twofold increase in rates of anti-coagulation (9.52% in 2005 vs 17.09% in 2014, P trend < 0.0001).

**Table 3:** Trends of hospitalization for atrial fibrillation admitted over the weekend 2005–2014

Years	2005	2006	2007	2008	2009	
Total number of hospitalization (weighted)	72,216	75,822	77,539	88,127	90,013	
Age in years						
18 to 34	2.33%	2.54%	2.36%	2.03%	2.21%	
35 to 49	8.16%	8.55%	8.44%	7.72%	7.78%	
50 to 64	22.09%	22.36%	21.89%	21.75%	22.29%	
65 to 79	37.35%	36.99%	37.27%	37.26%	37%	
Greater than 80	29.89%	29.4%	29.92%	31.06%	30.62%	
Indicator of sex						
Male	47.68%	47.99%	47.16%	47.22%	47.75%	
Female	52.32%	52.01%	52.84%	52.78%	52.25%	
Died during hospitalization						
Did not die	98.68%	98.87%	98.85%	98.88%	98.92%	
Died	1.32%	1.13%	1.15%	1.12%	1.08%	
Race						
White	85.34%	82.48%	81.55%	82.28%	82.23%	
Black	6.27%	7.71%	8.54%	7.6%	7.43%	
Hispanic	5.06%	6.25%	5.81%	5.39%	5.5%	
Asian or Pacific Islander	1.31%	1.31%	1.66%	1.55%	1.45%	
Native American	0.17%	0.46%	0.47%	0.58%	0.56%	
Other	1.85%	1.78%	1.96%	2.61%	2.83%	
Length of stay (LOS)						
0 to 3	62.84%	63.52%	63.99%	64.09%	63.81%	
4 to 6	26.36%	25.36%	25.32%	25.52%	25.81%	
7 to 9	5.59%	5.53%	5.79%	5.47%	5.54%	
10 to 12	2.91%	3.09%	2.98%	3.03%	2.95%	
Greater than 12	2.31%	2.49%	1.91%	1.9%	1.9%	
Mean LOS (days)	3.66	3.65	3.55	3.56	3.53	
Hospital location and teaching status						
Rural	16.83%	15.84%	16.66%	15.74%	14.83%	
Urban non teaching	49.85%	43.73%	45.48%	46.67%	47.46%	
Urban teaching	33.32%	40.43%	37.86%	37.59%	37.71%	
Comorbidities						
Alcohol abuse	4.05%	4.56%	4.42%	4.07%	4.48%	
Congestive heart failure	0.39%	0.36%	0.26%	0.31%	0.5%	
Depression	5.47%	6.18%	6.59%	7.57%	7.28%	
Diabetes with chronic complications	2.39%	2.24%	2.61%	2.71%	2.96%	
Hypertension (combine uncomplicated and complicated)	56.07%	59.4%	61.12%	63.5%	65.14%	
Liver disease	0.97%	0.97%	1%	1.38%	1.49%	
Obesity	7.03%	7.77%	9.53%	10.41%	11.93%	
Peripheral vascular disorder	4.66%	5.31%	5.59%	6.04%	6.01%	
Psychoses	1.58%	1.93%	1.74%	2.38%	2.13%	
Renal failure	5.02%	8.19%	9.51%	9.74%	11.33%	
Uncomplicated diabetes	17.43%	18.7%	19.37%	20.11%	21.02%	
Cardioversion rates	11.49%	12.21%	12.02%	12.55%	13.63%	
Anticoagulation	9.52%	10.55%	12.07%	12.13%	14.37%	
Cost of hospitalization (in US dollars)	6260.13	6807.54	7060.35	7287.86	7332.45	

Years	2010	2011	2012	2013	2014	P value (trend)
Total number of hospitalization (weighted)	89,600	97,140	97,174	95,089	92,220	
Age in years						
18 to 34	1.92%	1.99%	2.07%	1.71%	1.82%	< 0.0001
35 to 49	7.32%	6.76%	6.7%	6.69%	6.19%	< 0.0001
50 to 64	23.74%	23.03%	23.37%	22.9%	23.45%	< 0.0001
65 to 79	36.04%	37%	36.89%	37.91%	38.53%	0.0449
Greater than 80	30.88%	31.15%	30.89%	30.69%	29.93%	0.0177
Indicator of sex						0.0082
Male	48.36%	47.21%	47.93%	47.89%	48.13%	
Female	51.64%	52.79%	52.07%	52.11%	51.87%	
Died during hospitalization						< 0.0001
Did not die	99.01%	98.93%	99.04%	98.86%	99.06%	
Died	0.99%	1.07%	0.96%	1.14%	0.94%	
Race						
White	81.42%	82.03%	81.54%	81.62%	80.32%	< 0.0001
Black	8.81%	8.32%	8.72%	8.97%	9.03%	< 0.0001
Hispanic	5.77%	6.05%	5.64%	5.71%	6.25%	< 0.0001
Asian or Pacific Islander	1.44%	1.29%	1.42%	1.54%	1.66%	< 0.0001
Native American	0.71%	0.34%	0.44%	0.4%	0.43%	0.0102
Other	1.85%	1.96%	2.23%	1.76%	2.3%	< 0.0001
Length of stay (LOS)						
0 to 3	63.83%	65.81%	66.2%	66.11%	65.52%	< 0.0001
4 to 6	25.62%	24.62%	24.32%	24.16%	24.75%	< 0.0001
7 to 9	5.58%	5.02%	5.19%	5.1%	5.18%	< 0.0001
10 to 12	2.93%	2.78%	2.66%	2.62%	2.57%	< 0.0001
Greater than 12	2.04%	1.78%	1.64%	2.01%	1.98%	< 0.0001
Mean LOS (days)	3.58	3.41	3.4	3.44%	3.49	< 0.0001
Hospital location and teaching status						
Rural	14.83%	14.64%	13.92%	14.08%	11.73%	< 0.0001
Urban non teaching	46.38%	45.72%	42.51%	42.1%	30.63%	< 0.0001
Urban teaching	38.79%	39.63%	43.57%	43.82%	57.64%	< 0.0001
Comorbidities						
Alcohol abuse	4.74%	5.08%	5.35%	5.55%	5.83%	< 0.0001
Congestive heart failure	0.35%	0.4%	0.3%	0.36%	0.33%	0.4994
Depression	7.95%	8.84%	9.08%	9.2%	9.72%	< 0.0001
Diabetes with chronic complications	2.81%	3.5%	3.21%	3.48%	3.61%	< 0.0001
Hypertension (combine uncomplicated and complicated)	66.63%	68.5%	69.08%	69.91%	71.55%	< 0.0001
Liver disease	1.7%	1.76%	1.86%	1.79%	2.15%	< 0.0001
Obesity	12.08%	13.01%	15.2%	16.23%	17.62%	< 0.0001
Peripheral vascular disorder	6.46%	6.94%	7.11%	6.96%	7.55%	< 0.0001
Psychoses	2.41%	2.69%	2.68%	2.6%	2.94%	< 0.0001
Renal failure	12.71%	13.5%	14.28%	15.24%	16.15%	< 0.0001
Uncomplicated diabetes	22.1%	21.65%	22.97%	23.3%	23.95%	< 0.0001
Cardioversion rates	14.74%	14.55%	15. 7%	15.95%	17.34%	< 0.0001
Anticoagulation	15.18%	15%	15.48%	15.67%	17.09%	< 0.0001
Cost of hospitalization (in US dollars)	7568.3	7656.57	7719.1	8143.44	8265.78	< 0.0001

### Discussion

The main findings and trends noted in the current study of weekend AF hospitalizations are (1) improving trends in in-hospital mortality over 10 years from 2005 to 2014. (2) Weekend hospitalizations are associated with higher odds of in-hospital mortality. (3) Decreasing the mean length of hospital stay, and (4) increasing trends of utilization rates of cardioversion and anticoagulation.

The ‘weekend effect’ is a concern where the patients are thought to have worse outcomes when admitted to the hospital on a Saturday or a Sunday [11]. The first reports of the weekend hospitalizations having higher mortality appeared in the 1970s. Higher mortality and longer hospital LOS have been reported among AF hospitalizations during the weekend by Deshmukh et al. and Weeda et al. [3, 12] Subsequent study reported no difference in weekend and weekday AF in-hospital mortality [13].

In comparison to the prior studies, ours is the first study analyzing the trends of weekend AF hospitalizations. Our results match the results of Weeda et al. [3] where there is improved mortality among weekend hospitalization with AF. Though the lower utilization of cardioversion has been demonstrated through the years, the rates of cardioversion have significantly been improving, and at the same time, the in-hospital mortality has been decreasing during the same time period. This can be attributed to improved access to life-saving procedures. However, the overall utilization rates of cardioversion continue to be low among the weekend hospitalizations when compared to the weekday hospitalization. This is likely due to staffing issues, the availability of anesthesia, or coverage for a trans-esophageal echocardiogram at some institutions.

In the nationwide US practice, the weekend AF hospitalizations appear to have improved rates of in-hospital mortality, rates of cardioversion utilization and improved utilization of anticoagulation. However, the overall rates of in-hospital mortality continue to be poor in comparison to weekday hospitalizations. Further studies are required to identify the opportunities to improve AF weekend care.

## Limitations

Although our study has a large nationally representative database sample, these findings should be interpreted considering the following limitations. First, we identified our cases using ICD-9 discharge diagnosis codes, and details of the initial presentation (for example, emergency room visit) are not available, thereby, limiting the ability to confirm the diagnosis. Secondly, the NIS data does not provide information on important clinical predictors of outcomes such as the duration and the type of AF, left atrial diameter, the presence of thrombus in the left atrium and the baseline functional status, which can potentially influence the outcomes for in-hospital mortality. Third, given the description of ICD-9 codes in the database, it is not possible to differentiate pre-existing comorbidities from complications which have occurred during the hospitalization. Fourth, data regarding specific medical management such as anti-arrhythmic agents are not available in the NIS. And lastly, the diagnostic coding inconsistencies between weekends and weekdays also could not be ruled out. Given these limitations, it would require studies to have rigorous analysis having additional clinical information having a more consistent way of collecting data (such as using consistent diagnostic definitions) and analyzing outcomes considering all the above-mentioned factors [11].

## Data Availability

Data not available for publication per HCUP-AHR.

## Abbreviations

AF: atrial fibrillation
NIS: National Inpatient Sample
AHRQ: Agency for Health Care Quality and Research
ICD-9 CM: International Classification of Diseases-9, Clinical Modifications
LOS: length of stay
OR: odds ratio
